# Empowering Caregivers: Innovative Technology for Smarter, More Connected Care

**DOI:** 10.1093/geroni/igaf122.1135

**Published:** 2025-12-31

**Authors:** Walter Boot, Edie Sanders

## Abstract

Caregivers play a critical role in supporting older adults, yet they often face significant emotional, physical, and logistical challenges. This symposium presents innovative research on technology-based interventions designed to empower caregivers and optimize care delivery. Dr. Sara Czaja will provide an overview of digital solutions for family caregivers, discussing mobile applications, web-based platforms, and sensing systems and share findings on the feasibility, acceptability, and efficacy of these tools in improving caregiver outcomes. Taekyung Kim MS will present research on the adoption of AI-assisted care technologies among formal care providers, highlighting key factors such as social influence and effort expectancy that shape technology acceptance and use in real-world caregiving environments. Dr. Felipe Jain will discuss findings from an 8-week randomized controlled trial examining the impact of mentalizing imagery therapy (MIT) delivered via a mobile app on daily happiness and stress in family caregivers of people living with dementia. Dr. Xin Yao Lin will share results from a scoping review of app-based interventions for caregivers, identifying common design features, benefits, and research gaps that must be addressed to improve intervention effectiveness. Finally, Dr. Katherine Carroll Britt will present qualitative findings from caregivers and dementia care experts to inform the development of an AI chatbot designed to support cognitive care planning for individuals living with dementia and their care partners. By showcasing these diverse approaches, this symposium will explore the potential of digital innovations to create smarter, more connected caregiving solutions that enhance well-being and care quality for both caregivers and older adults.

